# Effect of Different Media on the Bactericidal Activity of Colistin and on the Synergistic Combination With Azidothymidine Against *mcr-1-*Positive Colistin-Resistant *Escherichia coli*

**DOI:** 10.3389/fmicb.2020.00054

**Published:** 2020-01-29

**Authors:** Maria Loose, Kurt G. Naber, Anthony Coates, Florian M. E. Wagenlehner, Yanmin Hu

**Affiliations:** ^1^Clinic for Urology, Pediatric Urology and Andrology, Justus-Liebig University Giessen, Giessen, Germany; ^2^Department of Urology, Technical University of Munich, Munich, Germany; ^3^Institute for Infection and Immunity, St George’s, University of London, London, United Kingdom; ^4^Helperby Therapeutics Ltd., London, United Kingdom

**Keywords:** colistin resistance, colistin, MIC/MBC, influence pH and cations, azidothymidine

## Abstract

Antimicrobial susceptibility testing (AST) performed according to defined guidelines is important to identify resistance and to predict the clinical success or failure of specific antibiotic therapy. However, these guidelines do not cover all physiological conditions that can have a tremendous impact on *in vivo* resistance. In this study, we tested the susceptibility of thirteen *mcr-1-*positive *Escherichia coli* strains against colistin, one of the last resort antibiotics for treating multi-drug resistant pathogens, in media recommended for ASTs as well as – physiologically more relevant – in human serum and artificial urine (AU). Minimal inhibitory concentration (MIC) values in heat-inactivated human serum were similar to those in cation-adjusted Mueller-Hinton broth (CAMHB), but reduced in native serum for almost all strains that could grow in this media. In AU MIC values for *mcr-1* positive *E. coli* were increased significantly up to 16-fold compared to that in CAMBH, which did not apply to the colistin-susceptible *E. coli* strains tested. Although different growth media could affect the MIC of colistin alone, their impact on the synergistic effect of the combination with the antiviral drug azidothymidine was minimal. The higher divalent cation concentration combined with acidic pH values is most likely responsible for the increased MIC values of the *mcr-1* harboring *E. coli* strains tested against colistin in AU compared to that in CAMHB. Antimicrobial susceptibility screening procedures for colistin using CAMHB only could lead to an underestimation of resistance under different physiological conditions. Therefore, not only pharmacokinetic but also pharmacodynamic studies in urine are as important as in serum or plasma.

## Introduction

Gram-negative bacteria, especially *Enterobacteriaceae* are the major cause of community and hospital acquired urinary tract infections (UTI). Due to a worldwide increasing number of multidrug-resistant (MDR) Gram-negative bacteria combined with a lack of development of new antibiotics, physicians are turning to older antibacterials to treat antibiotic-resistant infections. Thus, polymyxins like colistin, which was discarded because of toxicity concerns, are now seen as last line defense (World Health Organization [WHO], 2011).

Unfortunately, appreciable emergence of resistance to colistin has been shown recently (Paterson and Harris, 2016). Modifications of the lipid A moiety of lipopolysaccharide (LPS), which result in an inefficient binding of colistin, can be attributed either to a chromosome-encoded machinery or to the plasmid-transferred mobilized colistin resistance (*mcr-1*) gene (Baron et al., 2016; Cannatelli et al., 2016; Lui et al., 2016). MCR-1 is a member of the phosphoethanolamine transferase enzyme family catalyzing the addition of phosphoethanolamine to lipid A (Lui et al., 2016). Since the development of new antibiotics is decreasing, it is mandatory to find solutions to break the resistance to or enhance the antimicrobial activity of older antibiotics by co-administration of appropriate drugs, otherwise not used for antibiotic therapy. Recently, we have demonstrated that colistin in combination with the antiviral drug azidothymidine enhanced the colistin activity against colistin-resistant *Enterobacteriaceae* (Hu et al., 2018; Loose et al., 2018).

Antimicrobial susceptibility testing (AST) is important to predict the clinical outcome of an antibiotic therapy against particular pathogenic microorganisms. To ensure the reproducibility of the results, these tests are performed using guidelines established by the Clinical and Laboratory Standard Institute (CLSI) and the European Committee on Antimicrobial Susceptibility Testing (EUCAST). These guidelines recommend AST using cation adjusted Mueller-Hinton broth (CAMHB) as growth medium and define a bacterial inoculum of ∼5 × 10^5^ CFU/mL (Clinical and Laboratory Standards Institute, 1999, 2015). However, the susceptibility of bacteria to antibiotics are influenced by many factors such as bacterial inoculum size, composition of the medium (e.g., pH, ion concentrations) and host factors (e.g., serum factors) (Yang et al., 2014; Li et al., 2017). A recent study showed an increase of colistin Minimal inhibitory concentration (MIC) values for colistin-resistant strains by enhancing the calcium concentration in CAMHB (Gwozdzinski et al., 2018). But so far no data of MIC determinations of colistin in physiologically more relevant media such as urine or human serum have been published.

In this study we determined the minimal inhibitory and bactericidal concentrations (MIC/MBC) of colistin using different media, including artificial urine (AU) and human serum, with different inoculum size against *mcr-1-*positive as well as colistin-susceptible *E. coli* strains. In addition, we investigated whether these different media and inoculum sizes also affect the synergistic effect of azidothymidine on colistin antimicrobial activity.

## Materials and Methods

### Bacterial Strains

Thirteen clinical *Escherichia coli* isolates harboring the colistin resistance gene *mcr-1* as well as twelve colistin-susceptible *E. coli* strains (one reference strains, *E. coli* ATCC25922 and eleven clinical isolates) were included in this study (Supplementary Table S1).

### Determination of Minimal Inhibitory and Bactericidal Concentrations (MIC/MBC)

Minimal inhibitory concentration (MIC) and Minimal inhibitory bactericidal concentrations (MBC) determinations were performed according to the CLSI- and EUCAST-Standards (Clinical and Laboratory Standards Institute, 1999, 2015; Clinical laboratory testing, 2006). Broth microdilution assay was used for determination of the MIC of colistin sulfate (Sigma-Aldrich, Darmstadt, Germany) in CAMHB (Sigma-Aldrich), IsoSensitest broth (ISB) (Oxoid, Wesel, Germany), native and heat-inactivated (30 min at 56°C) human serum (Sigma-Aldrich), as well as AU, which contains [g/L] CaCl_2_ – 0.49, MgCl_2_ × 6H_2_O – 0.65, NaCl_2_ – 4.6, Na_2_SO_4_ – 2.3, Na_2_ citrate 2H_2_O – 0.65, Na_2_C_2_O_4_ – 0.02, KH_2_PO_4_ – 2.8, KCl – 1.6, NH_4_Cl – 1.0, Urea – 25.0, gelatine – 5.0 and Tryptone soya broth – 10.0; pH 6.1 (Stickler et al., 1999). Two final inocula were used: 10^5^CFU/mL and 10^6^ CFU/mL, confirmed by plating, ranged from 3.4–8.5 × 10^5^ CFU/mL and from 1–5 × 10^6^ CFU/mL, respectively. The MIC was defined as the lowest concentration inhibiting visible growth (OD_600_ < 0.1) after incubation at 37°C for 20 ± 2 h. For determination of MBCs, in a second step 3 μl were transferred onto IsoSensitest agar supplemented with 5% blood (Oxoid) using a one-time inoculator (Dr. Brinkmann Floramed, Nürtingen, Germany). The plates were incubated overnight at 37°C. The number of colonies subsequently grown was used to determine the bactericidal endpoint. MBC was defined as a >99.9% (>3-log) reduction of the initially inoculated colony counts (0.01% threshold for inoculum 10^5^/10^6^ CFU/mL: 1–2/10–12 colonies). MIC/MBC determinations were performed at least three times.

For chelating divalent cations in CAMHB and in AU 1 mM EDTA (ethylenediaminetetraacetic acid; Sigma-Aldrich) was added 30 min prior to colistin sulfate addition.

### Checkerboard Assays

A two-dimensional, two-agent broth microdilution checkerboard titration method was used to study the interaction between colistin sulfate and azidothymidine (AZT; Sigma-Aldrich) (Eliopoulos and Moellering, 1996). The final inoculum, confirmed by plating, ranged from 3.1–6.5 × 10^5^ CFU/mL or 1.0–5.3 × 10^6^ CFU/mL. After 20 ± 2 h of incubation at 37°C, the MIC and MBC of azidothymidine and colistin sulfate were determined. Checkerboard determinations were performed only once.

Interactions between azidothymidine and colistin sulfate were then evaluated using the fractional inhibitory/bactericidal concentrations indices (ΣFIC/ΣFBC) calculated as the sum of the FIC or FBC as follows:

ΣFIC=FIC+CSFICAZT

FIC – MIC of the substance in combination/MIC of the substance alone.

Correlation between ΣFIC/ΣFBC and the effect of the combination according to EUCAST definition (European Committee for Antimicrobial Susceptibility Testing [EUCAST] of the European Society of Clinical Microbiology and Infectious Diseases [ESCMID], 2000): Synergy − ≤ 0.5, additive >0.5–1, indifference >1 to < 2 and antagonism ≥2.

### Statistical Analysis

MIC and MBC values are reported as median values. High off-scale MIC results were converted to the next highest concentration and low off-scale results were left unchanged for comparisons. Concentration data were transformed to compensate for the doubling dilution series by log_2_ transformation prior to statistical analysis. Differences between MIC or MBC values were identified following analysis of variance (ANOVA) and *post hoc* analysis of significance for each of the variables. MIC/MBCs within ±1 log_2_ dilution (two-fold) were regarded as identical. Statistical calculations were performed by using Microsoft excel 2016.

## Results

### Minimal Inhibitory and Bactericidal Concentrations

The median of MIC determinations of colistin performed according to EUCAST and CLSI guidelines in CAMHB with an inoculum of 10^5^CFU/mL (Clinical and Laboratory Standards Institute, 1999, 2015; Clinical laboratory testing, 2006) revealed a range between 4–16 mg/L for the *mcr-1-*positive strains classifying them as colistin-resistant according to the EUCAST definition (there are no CLSI criteria for colistin and *Enterobacteriaceae*) (The European Committee on Antimicrobial Susceptibility Testing, 2017). Colistin MIC values for the non-*mcr-1* strains ranged from 0.25–1 mg/L classifying them as colistin-susceptible (Table 1). The median MBC values in CAMHB with an inoculum of 10^5^ CFU/mL hardly differ to the obtained MIC values for colistin-resistant as well as –susceptible strains (Table 1). Using IsoSensitest broth (ISB), another traditional medium used for antimicrobial testing, showed an slight colistin MIC increase for the colistin-resistant, but not the susceptible, strains. Since patients with symptomatic UTI usually show bacterial loads greater than 10^5^CFU/mL and since inoculum size can affect MIC values of antibiotics, we also performed MIC/MBC determinations with inocula of 10^6^ CFU/mL. There were no differences between median MIC and MBC values for the *mcr-1-*positive strains between using inoculum of 10^5^ and 10^6^ CFU/mL. In contrast, increasing inoculum led to increased MIC values in ISB for the colistin-susceptible strains (Table 1).

**TABLE 1:** Median MIC and MBC values of colistin in CAMHB and ISB with different inocula.

	**CAMHB 10^5^ CFU/mL**		**CAMHB 10^6^ CFU/mL**		**ISB 10^5^ CFU/mL**		**ISB 10^6^ CFU/mL**	
				
	**MIC [mg/L]**	**MBC [mg/L]**	**MIC [mg/L]**	**MBC [mg/L]**	**MIC [mg/L]**	**MBC [mg/L]**	**MIC [mg/L]**	**MBC [mg/L]**
***Colistin-susceptible E. coli***								
ATCC 25922	1	1	2	2	0.5	2	2	4
UTI89	0.5	0.5	1	1	0.5	1	1	2
CHD3	0.5	0.5	1	1	1	2	4	4
CHD4	0.5	0.5	1	1	0.5	2	2	2
CHD5	0.5	0.5	1	1	0.5	2	2	2
CHD6	0.5	0.5	1	2	0.5	2	1	2
CHD7	0.25	0.5	1	1	0.25	1	1	2
CHD8	0.5	0.5	1	1	0.5	1	2	2
CHD10	1	1	2	2	0.5	4	4	4
CHD11	0.5	0.5	1	1	0.5	2	2	2
CHD12	0.5	1	1	2	0.5	2	2	2
CHD16	1	1	1	1	0.5	1	2	2
Mean (range)	0.5 (0.25–1)	0.5 (0.5–1)	1 (1–2)	1 (1–2)	0.5 (0.25–0.5)	2 (1–4)	2 (1–4)^a^	2 (2–4)
***mcr-1-*positive *E. coli***								
Af23	8	8	8	8	16	16	8	8
Af24	8	16	16	16	32	32	16	64
Af31	6	16	8	16	16	16	16	32
Af40	8	16	8	32	16	16	16	32
Af45	8	16	8	16	32	32	16	64
Af48	6	8	8	16	16	32	16	16
Af49	8	16	8	8	32	32	16	32
CDF1	4	16	8	32	16	32	8	16
CDF2	16	16	16	32	32	32	32	32
CDF6	8	16	4	32	24	48	16	64
CDF8	8	16	8	64	16	24	16	32
S115	8	8	8	8	16	64	16	64
PS1	8	8	8	8	24	48	16	64
Median (range)	8 (4–16)	16 (8–16)	8 (8–16)	16 (8–32)	16 (8–32)	32 (8–64)	16 (8–32)	32 (8–64)

*≥4 fold (± 2 log_2_) difference with *p* < 0.0001: ^*a*^compared to 10^5^ CFU/ml in same media.*

Since all *E. coli* strains used were isolated mostly from blood and urine, we were interested to determine MIC values under these physiological conditions. Therefore, AU and human serum were used. The MIC values for the *mcr-1-*positive strains in AU were significantly increased up to16 fold (4 log_2_) compared to CAMHB for both inocula tested. In contrast, for colistin-susceptible *E. coli* strains the MIC values remained almost unchanged in AU compared to CAMHB (Table 2). Median MIC values in heated serum were almost equal to those in CAMHB for all strains tested. Furthermore increasing the inoculum size had no effect on the MIC values in AU and heated serum for the *mcr-1* positive *E. coli* strains tested, but resulted in a slight but significant increase of the MIC values of the colistin-susceptible strains in AU (Table 2). Not all of the strains used were able to grow constantly in native human serum. Therefore, MIC determinations in native serum were performed with lower number of strains. Median MIC values of the *mcr-1* containing *E. coli* (except for *E. coli* Af49), growing in native human serum, were clearly decreased as compared to those in CAMHB. Only the MIC value for *E. coli* Af49 was comparable in native serum (4 mg/L) to that in CAMHB (8 mg/L). In contrast, MIC values were only slightly reduced in native serum for colistin-susceptible strains with the lower inocula (Table 2).

**TABLE 2:** Median MIC values [mg/L] of colistin in artificial urine and human serum.

	**Artificial urine**		**Native serum**		**Heat inactivated serum**	
			
	**10^5^CFU/mL**	**10^6^CFU/mL**	**10^5^CFU/mL**	**10^6^CFU/mL**	**10^5^CFU/mL**	**10^6^CFU/mL**
***Colistin-susceptible E. coli***						
ATCC 25922	2	4	0.25	0.25	2	2
UTI89	0.5	2	nd	0.25	1	1
CHD3	0.5	2	nd	nd	1	1
CHD4	0.5	2	nd	nd	0.5	1
CHD5	0.25	2	0.125	1	1	1
CHD6	0.5	2	0.0625	0.5	0.5	2
CHD7	0.5	1	nd	nd	0.25	1
CHD8	0.5	2	0.0625	1	0.25	2
CHD10	1	4	0.5	2	2	4
CHD11	0.5	1	nd	nd	1	1
CHD12	0.5	4	0.125	2	1	4
CHD16	0.25	1	0.5	2	1	1
Median (range)	0.5 (0.25–2)	2 (1–4)^b^	0.13 (0.063–0.5)	1 (0.25–2)	1 (0.25–2)	1 (1–4)
***mcr-1-*positive *E. coli***						
Af23	64	64	0.0625	0.5	8	16
Af24	>64	64	nd	nd	16	16
Af31	64	64	nd	nd	8	8
Af40	>64	>64	0.125	nd	8	8
Af45	>64	64	nd	0.25	16	8
Af48	64	64	nd	nd	8	8
Af49	64	64	4	4	8	16
CDF1	64	64	0.5	1	8	8
CDF2	>64	>64	nd	nd	32	32
CDF6	>64	64	nd	0.5	16	16
CDF8	64	>64	0.5	0.25	8	8
S115	>64	>64	nd	0.5	16	16
PS1	>64	64	nd	0.5	8	8
Median (range)	>64 (64−>64)^a^	64 (64−>64)^a^	0.5 (0.063–4)	0.5 (0.25–4)	8 (8–32)	8 (8–32)

*≥4 fold (± 2 log_2_) difference with *p* < 0.0001: ^*a*^compared to corresponding inoculum in CAMHB; ^*b*^compared to 10^5^ CFU/ml in same media; nd – not definable.*

The *E. coli* strains tested showed a similar growth rate in AU compared to CAMHB (Supplementary Figure S1). Thus, a slower bacterial growth due to a lack of nutrients in the AU can be ruled out as a reason for the different MIC values. Since divalent cations are known to influence the activity of colistin (D’Amato et al., 1975; Gwozdzinski et al., 2018), we were interested in whether the increased MIC values in AU resulted from higher concentrations of Ca^2+^/Mg^2+^ (4.4/3.2 mM vs. 0.5/0.4 mM) compared to CAMHB. Supplementation of CAMHB with Ca^2+^ and Mg^2+^ to a final concentration of 3 mM increased the median MIC values eight-fold for all three *mcr-1-*positive strains tested (Table 3). In addition, adding of 1 mM EDTA to chelate divalent cations had no effect in CAMHB but in AU it diminished MICs 16-fold reaching values equal to CAMHB for *mcr-1-*positive strains. In contrast, cation-supplementation did not affect MIC values for the colistin-susceptible *E. coli* tested and EDTA addition led to no reduction of MIC values (Table 3). Furthermore, we determined the effect of difference in pH between AU (pH 6.1) and CAMHB (pH 7.3) by adjusting the pH to 7.3 and 6.1, respectively. For the *mcr-1-*positive strains, reducing the pH of CAMHB increased the MIC values, which was further enhanced by cation supplementation and was abolished by adding of EDTA comparable to AU. Changing of the pH or chelating cations by adding EDTA in AU did not affect MIC values of colistin for the colistin-susceptible *E. coli* strains (Table 3).

**TABLE 3:** Median MIC (range) in cation supplemented and/or pH adjusted CAMHB and artificial urine.

	**MIC [mg/L]**									
	**CAMHB pH 7.3**			**CAMHB pH 6.1**			**Artificial urine pH 6.1**		**Artificial urine pH 7.3**	
				
		**3 mM Ca^2+^/Mg^2+^**			**3 mM Ca^2+^/Mg^2+^**					
				
			** + 1mM EDTA**			**+1 mM EDTA**		** + 1 mM EDTA**		**+1 mM EDTA**
*CS-susceptible*	0.5	1	0.5	0.5	0.5	1	0.25	0.25	0.5	0.25
*E. coli* (*n* = 3)	(0.5–2)	(0.5–2)	(0.5–2)	(0.5–2)	(0.5–4)	(1–2)	(0.25–2)	(0.25–2)	(0.5–2)	(0.25–1)
*mcr-1-p*ositive	4	32^a^	32^a^	24^a^	>64^a,b^	6^b,c^	64^a^	4^d^	32^a^	16
*E. coli* (*n* = 3)	(4–8)	(16–64)	(8–64)	(16–32)	(64−>64)	(2–16)	(32−>64)	(2–16)	(4–64)	(2–32)

*≥4 fold (± 2 log_2_) difference with *p* < 0.01 ^*a*^ – compared to CAMHB pH 7.3. ^*b*^ – compared to CAMHB pH 6.1. ^*c*^ – compared to CAMHB pH 6.1 + 3 mM Ca^2+^/Mg^2+^. ^*d*^ – compared to artificial urine pH 6.1.*

### Checkerboard Assays to Determine the Combination Effect of Colistin and Azidothymidine

Recently we showed that a combination of colistin with the antiviral drug azidothymidine, which has also antibacterial properties (Elwell et al., 1987), was synergistic for the treatment of colistin-resistant *E. coli* (Hu et al., 2018; Loose et al., 2018). Since FIC indices calculations are based on MIC values measured by microbroth dilutions in CAMHB with an inoculum 10^5^ CFU/mL (Hu et al., 2018), we were interested to ask whether using a higher inoculum or different growth media could have an influence on the synergistic activity between colistin and azidothymidine. Using CAMHB and an inoculum of 10^5^ CFU/mL, the combination with azidothymidine reduced the colistin MIC_50_/MBC_50_ value for the *mcr-1-*positive strains by four-fold. Furthermore, based on the ΣFIC_min_ values the combination of colistin with azidothymidine was synergistic for 46% and additive for the remaining strains tested. The results were similar to the previous study (Hu et al., 2018). Based on the ΣFBC_min_ values synergistic activities were shown even for 69% of the tested strains (Table 4). Also with an increased inoculum and/or in ISB and AU, the MIC_50_ values of colistin were reduced by two to four-fold and the MBC_50_ values even by four to eight-fold with the combination of azidothymidine and colistin as compared to colistin alone (Table 4). Based on ΣFIC_min_/ΣFBC_min,_ however, the combination of colistin with azidothymidine was only slightly less synergistic in CAMHB/ISB with an inoculum of 10^6^ CFU/ml as compared to an inoculum of 10^5^ CFU/mL in CAMHB.

**TABLE 4:** MIC and MBC range. MIC/MBC_50_ and MIC/MBC_90_ values for colistin before and after combination with azidothymidine in different media/with different inoculum.

	**MIC (mg/L)**			**MIC (mg/L) in combination with azidothymidine**			**Results of checkerboard titration assays based on minimum ΣFIC**		
			
	**MIC range**	**MIC_50_**	**MIC_90_**	**MIC range**	**MIC_50_**	**MIC_90_**	**Synergistic**	**Additive**	**Indifferent**
CAMHB/10^5^ (*n* = 13)	4–8	4	8	0.25–2	1	2	6	7	0
CAMHB/10^6^ (*n* = 13)	4–16	8	16	0.5–8	4	8	2	11	0
ISB/10^6^ (*n* = 13)	8–32	32	32	1–32	8	8	4	7	2
AU/10^6^ (*n* = 4)	64−>64	64	>64	4–32	16	32	2	2	0

	**MBC (mg/L)**			**MBC (mg/L) in combination with azidothymidine**			**Results of checkerboard titration assays based on minimum ΣFBC**		
			
	**MBC range**	**MBC_50_**	**MBC_90_**	**MBC range**	**MBC_50_**	**MBC_90_**	**Synergistic**	**Additive**	**Indifferent**

CAMHB/10^5^ (*n* = 13)	4–16	16	16	0.25–8	2	4	9	4	0
CAMHB/10^6^ (*n* = 13)	4–64	32	64	1–8	4	8	7	5	1
ISB/10^6^ (*n* = 13)	8–64	32	64	1–32	8	8	9	3	1
AU/10^6^ (*n* = 4)	128–256	128	256	8–32	16	32	4	0	0

## Discussion

According to EUCAST and CLSI guidelines, our study demonstrated that all the *mcr-1-*positive *E. coli* strains tested are resistant to colistin as confirmed in previous studies (Poirel et al., 2016; Hu et al., 2018; Loose et al., 2018). We showed for the first time, that MIC values for the *mcr-1-*positive strains, but not colistin-susceptible strains, significantly increased up to 16-fold in AU compared to CMHB. This increased resistance depends most likely on the cationic concentrations and pH of the medium.

It is well known that increasing concentrations of divalent cations antagonize the activity of colistin (D’Amato et al., 1975). In addition our data showed that higher concentrations of divalent cations in AU increased the MIC values of *mcr-1* positive colistin-resistant strains. Since MIC values of colistin-susceptible strains were not affected, it can be assumed that increased cation concentrations do not generally inhibit the antimicrobial activity of colistin. Recently, Gwozdzinski et al. showed an increase of colistin MIC values in calcium enhanced (5 mM) CAMHB compared to normal CAMHB for *mcr-1* harboring colistin-resistant *E. coli*, but not colistin-susceptible *Enterobacteriaceae* strains. Furthermore, they showed that MIC values increased in calcium enhanced media after a colistin-susceptible strain was transformed with different *mcr-1*-containing plasmids (Gwozdzinski et al., 2018). This supports our assumption that increased cation concentrations influence the *mcr-1* mediated colistin resistance. Despite the relatively small number of strains used in this study, the *mcr-1* positive *E. coli* strains represent a heterogeneous group including different plasmids, sequence types and source of isolation (Supplementary Table S1). Irrespective of these differences, each individual *mcr-1* positive strain tested, but none of the colistin-susceptible strains, showed a marked MIC increase of 6–16 fold in AU compared to CAMHB, leading to the assumption that the calcium-induced *mcr-1* mediated colistin resistance is independent of the different plasmids groups or sequence types. This was also indicated by Gwozdzinski et al. (2018). Since plasmid-encoded *mcr-1* polymyxin resistant has also been identified in other *Enterobacteriaceae* as *Klebsiella pneumoniae* and *Enterobacter cloacae*, a calcium-induced resistance could also be assumed for these strains. However, this must be further investigated, as well as a possible cation- and pH-dependence of the other plasmid-borne colistin resistance genes, such as *mcr-2*, *mcr-3*, and *mcr-4.*

Resistance of the *mcr-1-*positive strains is mediated by 4′-phosphoethanolamine modification of the lipid A on LPS masking the negatively charged phosphate groups on the bacterial surface, which are involved in interactions with colistin. This modification is catalyzed by the MCR-1 enzyme (Pristovsek and Kidric, 1999; Lui et al., 2016). On the one hand, divalent cations may act as cofactors during lipid A modification as for a Ca^2+^-induced phosphoethanolamine transferase in *E. coli* (Kanipes et al., 2000). On the other hand, increased cation levels could result in positively charged modified LPS. Furthermore, acidification of CAMHB hand in hand with increasing cation concentrations resulted in increased MIC values for the *mcr-1-*positive strains. Thus, the lower pH of the AU could additionally promote the reduction of the net negative charge of lipid A, effectively diminishing the electrostatic colistin-bacteria interaction. In addition, low pH may enhance the phosphoethanolamine addition to lipid A as was shown for *Cronobacter sakazakii* (Liu et al., 2016). Concentrations of colistin, necessary to eliminate *mcr-1-*positive strains, are much higher under physiologically relevant conditions in AU. Assumptions that antibiotic concentrations reached in urine are high enough to eradicate even resistant pathogens are often based only on pharmacokinetic, but not pharmacodynamic results using MIC values obtained in CAMHB only. Pharmacokinetic parameters, like AUC (area under the concentration-time curve)/MIC and C_max_ (maximum concentration of the drug in urine)/MIC ratios will often be much less favorable under physiologically relevant conditions. Therefore, not only pharmacokinetic but also pharmacodynamic studies in urine are as important as in serum or plasma.

Antibiotic susceptibility increases notably in native serum for almost all strains, including those that are able to grow in serum. Serum contains more than thirty proteins of the complement system, which is the first line of defense and is essential for a rapid elimination of invading pathogens. An activated complement cascade triggers the formation of a ring-structured pore (the membrane-attack complex), which increases the permeability of the bacterial membrane (Walport, 2011). Weakening the bacterial membrane integrity by the complement system, even without killing the bacteria, could enhance the effectiveness of colistin. Thus, lower concentrations of colistin are usually necessary to kill bacteria in native serum. On the other hand, lipid A modifications to evade complement system could encourage the binding of colistin leading to an increased antimicrobial activity. For instance, one strategy to evade the attack of the complement system and thus being able to grow in serum is a switch to less acylated lipid A structures in the LPS (Matsuura, 2013). Coincidently, it was shown that *K. pneumoniae* strains with under-acylated lipid A are more susceptive to polymyxin B binding (Velkov et al., 2013). Susceptibility of colistin in heat inactivated human serum, by which the complement system is destroyed, did not differ from those in CAMHB.

Results of our checkerboard testing confirmed the recent findings (Hu et al., 2018), namely that the combination of colistin with azidothymidine can reduce the MIC of colistin by about 2 to 32 fold for *mcr-1-*positive *E. coli* in CAMHB. The antibacterial activity of azidothymidine can be traced back to the inhibition of the replicative DNA-synthesis after incorporation of azidothymidine-triphosphate (Olivero, 2016). The membrane permeabilization by colistin (Katz et al., 2003) could increase the entry of azidothymidine into the bacterial cells. The beneficial effect of this combination is even more pronounced when testing the effect for the MBCs. Therefore, additional evaluation of MBC data could be an advantage to show actual bacterial killing. Although the increase of the inoculum size or changing the growth media to ISB or AU affected the MIC of colistin alone, they only had a small influence on the synergistic effect of the colistin and azidothymidine combination. It should be noted, however, that the colistin MICs/MBCs in AU, even when reduced by the combination with azidothymidine, are still higher in AU than those of colistin alone in CAMHB.

## Data Availability Statement

All datasets generated for this study are included in the article/Supplementary Material.

## Author Contributions

ML performed all the experiments and wrote the manuscript, with close cooperation and supervision of FW and considering critical comments from all coauthors. KN, YH, and AC made substantial contributions to the conception and design, and all authors contributed to analysis and interpretation of the data. YH and AC were the co-inventors of the antibiotic resistance breaker technology, and in particular of the combination of azidothymidine and colistin/CMS (patents issued), first to test this combination against highly resistant Enterobacteriaceae (AAC 2018, pii: AAC.01630-18. 10.1128/AAC.01630-18), and originated the concept and performed the background work upon which this work is based. YH and AC revised the manuscript critically for important intellectual content and gave final approval of the revised version of the manuscript.

## Conflict of Interest

ML reports grants from Helperby Therapeutics Ltd., during the conduct of the study. KN reports personal fees from Adamed, personal fees from Allecra, personal fees from Apogepha, personal fees from Bionorica, personal fees from Enteris Biopharma, personal fees from Galenus, personal fees from GlaxoSmithKline, personal fees from Hermes, personal fees from Leo, personal fees from Medice, personal fees from MerLion, personal fees from MSD SHARP& DOHME, personal fees from Paratek, personal fees from Roche, personal fees from Rosen, personal fees from Saxonia, personal fees from Vifor, outside the submitted work. AC as Chief Scientific officer of Helperby Therapeutics Ltd., was involved in designing the study as well as interpreting the data. FW reports personal fees and other from Achaogen, personal fees from AstraZeneca, personal fees from Bionorica, other from Enteris BioPharma, other from Helperby Therapeutics Ltd., personal fees from Janssen, personal fees from LeoPharma, personal fees from MerLion, personal fees from MSD, personal fees from OM Pharma/Vifor Pharma, personal fees from Pfizer, personal fees from RosenPharma, personal fees and other from Shionogi, personal fees from VenatoRx, personal fees from GSK, outside the submitted work. YH reports grants and other from Helperby Therapeutics Ltd., during the conduct of the study. In addition, YH and AC have a patent “Zidovudine combination therapies for treating microbial infections” issued.
