# Supplementary material for: Effect of Different Media on the Bactericidal Activity of Colistin and on the Synergistic Combination With Azidothymidine Against mcr-1-Positive Colistin-Resistant Escherichia coli
Source: Front Microbiol. 2020 Jan 29;11:54. doi: 10.3389/fmicb.2020.00054 (PMC7000358; doi:10.3389/fmicb.2020.00054)
Supplement: Supplementary file 1 [file Data_Sheet_1.docx]

TABLE S1 Features of the *mcr-1* positive *E. coli* isolates

| **Strain** | ***mcr-1* positive plasmid type/ plasmid size (ca. kb)** | **Sequence type** | **Source** | **Co-Resistances** | **Co-resistance gene** | **Country of Isolation** | **Reference** |
| --- | --- | --- | --- | --- | --- | --- | --- |
| *Af23* | IncI2/ 70 | 10 | Human  blood | AMX-CIP-SXT-TET | *bla*_TEM-1_ | ZA | [1] |
| Af24 | IncI2/ 65 | 1007 | Human  pus | AMX-CIP-SXT-TET-CHL | *bla*_TEM-1_ | ZA | [1] |
| Af31 | IncHI2/ 150 | 624 | Human  urine | AMX-CIP-SXT-TET-CHL | - | ZA | [1] |
| Af40 | IncI2/ ND | 57 | Human  wound | AMX-CTX-CIP-SXT-TET-CHL-FOS | *bla*_CTX-M-55_ + *fosA3* + *tetR* | ZA | [1] |
| Af45 | IncI2/ 70 | 101 | Human  urine | SXT-TET | - | ZA | [1] |
| Af48 | Incx4/ 30 | 624 | Human  urine | AMX-CEF-CIP-SXT-TET-KAN | *bla*_CMY-2_ | ZA | [1] |
| Af49 | ND/ ND | 226 | Human  urine | AMX-CTX-CIP-SXT-TET-CHL-FOS | *bla*_CTX-M-55_ + *fosA3* + *tetR* | ZA | [1] |
| CDF1 | IncFIB/ 90 | 3077 | Human  blood | AMX-SXT-TET-CIP-GEN | *bla*_TEM-1_ | CH | - |
| CDF2 | IncFIB/ 90 | 131 | Human  blood | AMX-CTX-NAL-SXT-TET-GEN | *bla*_TEM-1_+  *bla*_TEM-52_ | CH | - |
| CDF6 | IncHI2/ 150 | 446 | Human  urine | AMX-CTX-CIP-SXT-TET | *bla*_CTX-M_ | CH | - |
| CDF8 | Incx4/ 30 | 167 | Human blood | AMX-CTX-CIP | *bla*_CTX-M_ | CH | - |
| S115 | Incx4/ 30 | 23 | Human  Urine | IPM-CIP-CHL-TET-SXT | *bla*_VIM-1_ + *bla*_CMY-2_ + *floR* | CH | - |
| PS1 | IncHI2/ ND | new | animal  stool | AMX-TET-SXT | - | FR | - |
|  |  |  |  |  |  |  |  |
| *ATCC25922* | - |  |  | - | - | Obtained from ATCC | |
| *UTI89* | - | 95 | Human  Urine | - | - | Obtained from DSMZ | |
| *CHD3* | - | 73 | Human  Urine | - | - | DE |  |
| *CHD4* | - | 1064 | Human  Urine | - | - | DE |  |
| *CHD5* | - | 131 | Human  Urine | - | - | DE |  |
| *CHD6* | - | 141 | Human  Urine | - | - | DE |  |
| *CHD7* | - | 2020 | Human  Urine | AMX | *bla*_TEM-1B_ | DE |  |
| *CHD8* | - | 5640 | Human  Urine | AMX | *bla*_TEM-1B_ | DE |  |
| *CHD10* | - | 126 | Human  Urine | - | - | DE |  |
| *CHD11* | - | 69 | Human  Urine | - | - | DE |  |
| *CHD12* | - | 446 | Human  Urine | - | - | DE |  |
| *CHD16* | - | 131 | Human  Urine | AMX-CIP-CTX-GEN | *bla*_CTX-M-15_ + *bla*_TEM-1B_ | DE |  |

AMX – Amoxicillin; CHL – Chloramphenicol; CIP – Ciprofloxacin; CTX – Cefotaxime; FOS – Fosfomycin; GEN – Gentamicin; IMP – Imipenem; KAN – Kanamycin; NAL – Nalidixic acid; SXT – Trimethoprim/Sulfamethoxazole; TET - Tetracycline

ZA – South Africa; CH – Switzerland; FR – France; DE – Germany, ND – not determined

**

**

**Figure S1. Mean growth curves of four *mcr-1* positive *E. coli* strains in artificial urine and CAMHB**

Bacteria were inoculated with ~ 1 x 10^6^ CFU/mL in artificial urine (AU) and CAMHB and were incubated at 37°C, 180rpm for 24h. After different time points samples were taken, serial diluted and plated on CAMHB-agar plates for CFU/mL determination. Shown are mean values +/- standard deviation of four *mcr-1* positive strains.

**References:**

1. Poirel L, Kieffer N, Brink A, Coetze J, Jayol A, Nordmann P. 2016. Genetic features of MCR-1-producing Colistin-resistant Escherichia coli isolates in South Africa. *Antimicrob Agents Chemother* 60:4394-7
